# It Is Time to Close the Gap in Cancer Care

**DOI:** 10.1200/GO.22.00429

**Published:** 2023-01-27

**Authors:** Jeff Dunn

**Affiliations:** ^1^ Union for International Cancer Control, Geneva, Switzerland; ^2^ University of Southern Queensland, Division of Research and Innovation, Queensland, Australia

Cancer is a leading cause of death in every country worldwide.^1^ In 2020, almost 10 million people died from cancer, a number which is expected to rise to 16.3 million by 2040.^2^ Alongside this, cancer incidence continues to grow, driven by an aging and growing population and changes in the prevalence and distribution of cancer risk factors. Specifically, over the next 2 decades, the number of new cancer cases will rise more than 50% to 30.2 million.^2^ While the overall burden grows, so do inequities in who can access cancer services, who has a greater chance of survival, and what the financial and social impacts of a cancer diagnosis are for individuals, their families, and communities. Addressing these inequities is a critical challenge for the cancer community, but it is the one that we must tackle to achieve higher quality of life and better outcomes for all.

Critically, there is substantial global diversity in cancer mortality, incidence, prevalence, and risk factors on the basis of national social and economic development.^1,3^ Currently, half of all cases and 58.3% of cancer deaths occur in Asia where almost 60% of the population resides. Europe accounts for almost one fifth of cancer cases and deaths despite representing < 10% of the global population. Similar to Asia, the share of cancer deaths versus incidence is higher in Africa because of different distributions of cancer type and higher case fatality rates.^1,4^ On the basis of the four-tier Human Development Index (HDI),^1,3^ cancer incidence rates are up to three times higher in very high-HDI countries compared with low-HDI countries, but the relative magnitude of increase is most notable in low- (95%) and medium-HDI countries (64%).^1,3^ In addition, many low- and medium-HDI countries are seeing a marked increase in the prevalence of risk factors, such as smoking, poor diet, obesity, and physical inactivity, which more commonly occur in high-HDI countries.^1,4^ Unfortunately, these countries may be least well equipped to address the future impact from these risk factors as services for noncommunicable diseases, including cancer, are very limited and not able to meet the growing need.

These trends and inequalities are the result of multiple factors reflecting socioeconomic development, culture, environment, geographic location, sex, and distribution of resources and services and are evident both within and between countries.^4,5^ For example, cancer survival continues to improve in very high-HDI countries, likely as a direct result of technical advances that facilitate earlier diagnosis and improved treatment and major policy reforms that support better patient outcomes.^6^ By contrast, in low-HDI countries, limited screening facilities, poor public health service infrastructure, poor health literacy and insufficient human and financial resources for cancer diagnosis, treatment, and management all contribute to a higher prevalence and lower survival rates.^7,8^

Within countries, there are also stark differences in cancer prevalence and outcomes on the basis of socioeconomic and geographic status, age, sex, and social and cultural factors. A recent landmark report on cancer in Scotland^9^ shows that cancer death rates are 74% higher in the most disadvantaged populations compared with the least. Scotland has the highest proportion of cancers caused by preventable risk factors in the United Kingdom, and almost 5,000 cases per year are directly attributable to inequalities across the cancer pathway.^9^ In the United States, African American/Black people have higher mortality than any other racial/ethnic groups for most cancers, driven primarily by lower socioeconomic status and unequal access to care.^10^ In Australia, overall incidence, cancer-related mortality, and cancer burden are significantly higher among socioeconomically disadvantaged groups, with the least advantaged quintile experiencing 34% more cancer-related mortality on average compared with their most advantaged counterparts.^11^ This is particularly evident for men with prostate cancer,^12^ a finding that is also reflected globally, with strong evidence that men with prostate cancer living in rural or disadvantaged areas have higher risk of advanced disease and mortality and poorer survival outcomes and access or use of medical services compared with men in urban/affluent areas.^12,13^

Another striking gap within countries is the inequalities in cancer outcomes faced by indigenous peoples in high-HDI countries.^14^ In Australia, the mortality is 39% higher for all cancers combined in First Nations People compared with other Australians, with the inequality in mortality widening by 82 deaths per 100,000 in the past 25 years.^15^ In New Zealand, the Māori continue to experience poorer survival than non-Māori New Zealanders for 23 of 24 cancers, with disparities up to 156% when adjusted for age and sex.^16^

So, how then are we to take global action? World Cancer Day, an initiative of the Union for International Cancer Control (UICC), originated on February 4, 2000, at the World Summit Against Cancer for the New Millennium in Paris. The key aims of this day are to raise cancer awareness, foster cancer education, and press governments and individuals across the world to take action against this disease. Since its inception, World Cancer Day has grown into a global movement with more than 900 activities in 105 countries in 2022. Importantly, 2022 saw the launch of the 3-year Close the Care Gap campaign, which shines a spotlight on the issue of equity in cancer care. In its first year, the Close the Care Gap campaign focused on understanding inequities in cancer care globally; 2023 sees the focus move to building stronger alliances and innovative new collaborations; the final year will issue a challenge to those in power to eliminate health inequities by addressing their root causes and supporting access to quality health services through specific actions focused on reaching the most in need.

The Close the Care Gap campaign directly aligns with the Sustainable Development Goals, which were adopted globally at the 2015 UN General Assembly. This agenda built on the Millennium Development Goals (2000-2015) and, to our knowledge, was the first time that the global development agenda recognized that noncommunicable diseases, including cancer, constitute a major health and development challenge, which affects all facets of sustainable development. UICC worked closely with the Non-Communicable Diseases Alliance in the lead up to the UN Summit in September 2015 to position noncommunicable diseases within the Sustainable Development Goals. The inclusion of Target 3.4 “By 2030, reduce by one third premature mortality from noncommunicable diseases through prevention and treatment, and promote mental health and wellbeing” and Target 3.8 “achieve universal health coverage (UHC), including financial risk protection, access to quality essential health care services, and access to safe, effective, quality, and affordable essential medicines and vaccines for all” is the important milestone for the noncommunicable disease community.

As the President of UICC, I urge the world to come together to inspire change and mobilize action against disparities in cancer care and outcomes. The sheer global diversity of cancer survivorship and the experiences of different people reinforce the need to enhance our efforts around cancer control toward better addressing the multitude of cancer inequalities evident in countries around the globe. This will require long-term, sustained, global efforts with all major health partners joining forces.

The reality today is that who you are and where you live could mean the difference between life and death. The new 3-year World Cancer Day Close the Care Gap campaign seeks to inspire change and spur action to address these disparities.^5^ In September this year, there will be a second UN High Level Meeting on UHC. This meeting provides a critical advocacy opportunity for the inclusion of cancer care within national UHC plans. Taking action to strengthen health care infrastructure and develop equitable systems that support care, the training and retention of health personnel, development and implementation of national cancer control plans, support for cancer registries and data repositories on risk factors and cancer treatment outcomes, and access to essential cancer medicines are important steps toward developing a strong cancer care system for all who need it. We should not waste this opportunity to advocate to our governments that quality cancer services must be integrated into UHC plans to improve cancer outcomes and reduce the huge out-of-pocket spending that many people have in paying for their cancer care.

Finally, when thinking about the care gap, do not lose sight of the individual patient, the person whose welfare provides us with purpose and whose quality of life must remain central to our efforts. I call you to act for yourself, for your own family, and for our global community. Each of us has a role to play, and together, we can close the care gap.
